# Role of central muscarinic cholinergic receptors in the formalin-induced pain in rats

**DOI:** 10.4103/0253-7613.55205

**Published:** 2009-06

**Authors:** Ali Mojtahedin, Esmaeal Tamaddonfard, Ali Zanbouri

**Affiliations:** Division of Physiology, Department of Basic Sciences, Faculty of Veterinary Medicine, Urmia University, Urmia, Iran

**Keywords:** Atropine, brain, formalin-induced pain, physostigmine, rats

## Abstract

**Objectives:**

In the present study, central effects of physostigmine and atropine have investigated in the formalin-induced pain in rats.

**Materials and Methods:**

In conscious rats implanted with an intracerebroventricular (i.c.v.) cannula, the effects of i.c.v. injection of physostigmine and atropine were investigated on the formalin test in the rat. Formalin test was induced by subcutaneous (s.c.) injection of formalin (50 μl, 1%) in ventral surface of left hind paw, and durations of licking and biting of the injected paw were measured in 5-min blocks for 1 h.

**Results:**

Formalin produced a biphasic response (first phase: 0–5 and second phase: 15–40 min) in durations of licking and biting of the injected paw. Physostigmine at doses of 2.5, 5 and 10 ug significantly (*P* < 0.05) attenuated both first and second phases of pain response. Atropine (5 and 10 ug), used alone, produced no significant effect on pain, but pretreatment with atropine (10 ug) significantly (*P* < 0.05) blocked antinociception induced by physostigmine (5 ug).

**Conclusion:**

These results indicate that i.c.v. physostigmine can affect both neurogenic and inflammatory phases of formalin-induced pain through a mechanism in which the muscarinic cholinergic receptors are involved.

## Introduction

Acetylcholine, through its muscarinic and nicotinic receptors, plays pivotal roles in a diverse array of physiological processes such as learning, memory, anxiety, epilepsy, attention, cognition and consciousness.[1–4] The activity of acetylcholine is controlled through enzymatic degradation by acetylcholine esterase in the sites of cholinergic transmission.[5] There are some evidences, which suggest that cholinesterase inhibitors, such as neostigmine and physostigmine, are involved in modulation of pain. Intraarticular and intrathecal injections of neostigmine produced analgesia in inflamed knee-joint model of pain in rats, and this effect was reversed by intrathecal pretreatment with atropine.[6] Moreover, intrathecal administration of neostigmine increased withdrawal threshold in tactile allodynia using von Frey filaments in the L_5_–L_6_ spinal nerves tight ligature model of neuropathic pain in rats.[7] Subcutaneous injection of physostigmine produced antinociceptive effect in the spinal nerve ligature model of neuropathic pain in rats.[8] In the tail immersion test of nociception in the rat, an antinociceptive effect of subcutaneous (s.c.)-injected physostigmine was reported.[9] It has been reported that intraperitoneal injection of physostigmine produced an antinociceptive effect in both tail flick and acetic acid-induced writhing tests in the mouse.[10] Intrathecal injection of physostigmine inhibited bradykinin-induced bioelectric activity in spinal ventrolateral tract in rats.[11] The antinociceptive effect of intrathecally administered acetylcholine esterase inhibitors such as neostigmine, physostigmine and edrophonium have been reported in the formalin test in rats.[12–15]

Physostigmine as a centrally acting cholinesterase inhibitor and atropine as a centrally acting muscarinic antagonist,[16] were used in the study of pain mechanisms by peripheral and intrathecal routes of administration.[8–15] There are no reports showing the effect of intracerebroventricular (i.c.v.) injection of physostigmine and atropine on the formalin-induced pain in rats and mice. Therefore, the aim of the present study was to investigate the direct supraspinal effects of physostigmine, a cholinesterase inhibitor and atropine, a muscarinic cholinergic receptor antagonist applied by i.c.v. route on formalin-induced pain in the rat.

## Materials and Methods

### Animals

Healthy adult male Wistar rats, weighing 220–250 g were used in this study. Rats were maintained in polyethylene cages with food and water available *ad libitum*, in a laboratory with controlled ambient temperature (23 ± 0.5°C) and under a 12-h light–dark cycle (lights on from 07:00 h). Six rats were used in each experiment. Experiments were carried out between 12:00 and 16:00 h. The experimental protocol was approved by the Laboratory Animal Care and Use Center of the Faculty of Veterinary Medicine of Urmia University.

### Drugs

Drugs used in the present study included physostigmine (Eserine) and atropine sulfate (Sigma – Aldrich, Steinheim, Germany). All drugs were dissolved in normal saline.

### Surgery

After a 15-day adaptation period, each rat was anesthetized with a mixture of ketamine hydrochloride 10% (80 mg/kg) and xylazine hydrochloride 2% (10 mg/kg) injected intraplantar (i.p.), and then placed in a stereotaxic apparatus (Stoelting, Wood Lane, IL, USA). The bone was exposed after incision of the skin. A hole was drilled in the bone and a 22 gauge, 12 mm stainless-steel guide cannula was inserted in the right lateral ventricle of the brain. The tip of the cannula was aimed at the following coordinates: 0.8 mm posterior to the bregma, 2 mm lateral to the midline and 4 mm below the top of the skull.[17] The cannula was then fixed to the skull using three screw and dental acrylic. A 12.5 mm stylet was inserted in the cannula to keep it patent prior to injection. Animals were allowed a 10-day recovery period before initiation of the experiments.

### Drug injection

For i.c.v. injections of normal saline (control), physostigmine and atropine, a 28 gauge, 12.5 mm injection needle was attached to a 30 cm polyethylene tube fitted to a 5 μl Hamilton syringe. Then, the rat was restrained by hand, the stylet was withdrawn and the injection needle was inserted into the guide cannula. The volume of the solutions to be injected into the lateral ventricle was 1 μl and the injection was slowly made over a period of 30 s. The injection needle was left in place for an additional 15 s to aid the diffusion of drug into cerebrospinal fluid (CSF). Atropine and physostigmine were adminisrered i.c.v. 10 and 5 min before induction of formalin pain, respectively.

### Formalin test

Formalin test was used for induction of pain. Before rats were pain tested, they were placed in a Plexiglass observation chamber (30 × 30 × 25 cm) for 30 min on three successive days to minimize stress-activated pain suppressive mechanisms.[18] The formalin test was applied as follows. Fifty microliters of 1% formalin was injected subcutaneously into the ventral surface of right hind paw using a 29-gauge injection needle.[19] The rat was then placed in the observation chamber with a mirror mounted at 45° beneath the floor to allow an unobstructed view of the paw. The time spent licking and biting the injected paw was taken as a measure of nociceptive response and was recorded in 5-min intervals for 1 h. In the present study, data collected between 0- and 5-min post-formalin injection represented phase one (early phase) and data collected between 15 and 45 min after injection of formalin represented phase two (late phase).

### Cannula verification

During the surgery and before i.c.v. injections, a rise in the CSF through the cannula was observed. For additional confirmation of the placement of the cannula in the lateral ventricle of the brain, at the end of experiments, the rats were i.c.v. injected with 10 μl methylene blue, and they were then deeply anesthetized with a high dose of ether and decapitated. The brains were removed and placed in a formaldehyde (10%) solution. After 24 h, the brains were sliced into 1 mm slices and the placement of the tip of the cannula and distribution of the dye in the lateral ventricle were confirmed using a loop. Data from rats with an incorrect placement of the cannula were excluded from the analysis.

### Statistical analysis

To evaluate significant differences among treated groups, one-way analysis of variance (ANOVA) and Duncan's test were applied. All values are expressed as the mean ± SEM. Statistical significance was set at p < 0.05.

## Results

**Figure 1:**
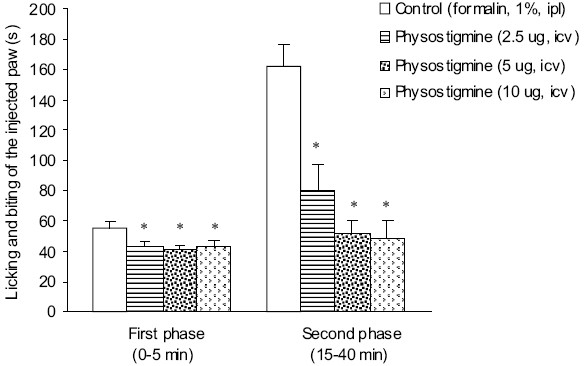
Effect of i.c.v. injection of physostigmine on the formal-ininduced pain in rats. Each column represents mean ± SEM (n = 6). *P < 0.05 vs. control (one-way ANOVA followed by Duncan's test). i.p.l.: intraplantar, i.c.v.: intracerebroventricular

**Figure 2:**
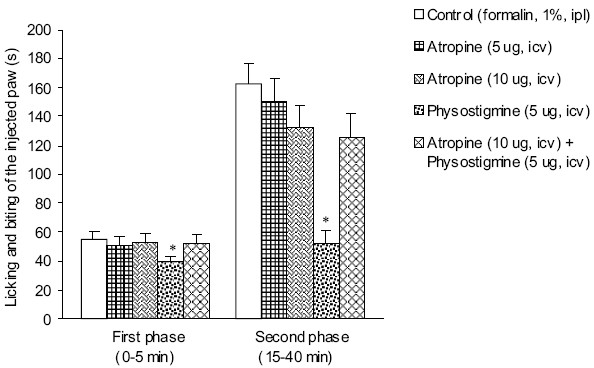
Effect of i.c.v. injection of atropine on physostigmine-induced changes in formalin-induced pain in rats. Each column represents mean ± SEM (n = 6). *P < 0.05 vs. other groups (one-way ANOVA followed by Duncan's test). i.p.l.: intraplantar, i.c.v.: intracerebroventricular

The durations of licking and biting of the injected paw in first and second phases after intraplantar injection of normal saline were 3.0 ± 0.9 and 1.2 ± 0.5 s, respectively. The results obtained from i.p. injection of normal saline are not presented in the figures. Intraplantar injection of formalin 30 min after intracerebroventricular injection of normal saline produced a biphasic (54.7 ± 4.8 s in 0–5 min and 162.5 ± 14.0 s in 15–40 min) response in duration of licking and biting of the injected paw [Figures 1 and 2].

Intracerebroventricular injection of physostigmine at doses of 2.5, 5 and 10 μg significantly (*p* < 0.05) decreased duration of licking and biting of the injected paw in both first and second phases of formalin-induced pain. No significant difference was observed among the different doses of physostigmine in first phase of pain, but in the second phase of pain, suppressive effect of physostigmine at doses of 5 and 10 μg was significantly (*p* < 0.05) higher than that of 2.5 μg. There was no significant difference observed between effects of 5 and 10 μg of physostigmine in second phase of pain [Figure 1].

Intracerebroventricular injection of atropine at doses of 5 and 10 μg, used alone produced no significant effect in both first and second phases of formalin-induced pain response, but i.c.v. pretreatment with atropine (10 μg) significantly prevented suppressive effect of i.c.v. injected physostigmine (5 μ) in both first and second phases of pain induced by formalin [Figure 2].

## Discussion

In the present study, it was found that i.c.v. injection of physostigmine produced antinociception. In addition, physostigmine-induced antinociception was blocked by atropine pretreatment. These indicate that acetylcholine esterase inhibitor, physostigmine, through muscarinic cholinergic receptors, may have a role in central modulation of pain. Physostigmine is a major alkaloid found in the seeds of the fabaceous plant ***Physostigma venenosum***, and is a powerful and reversible acetylcholine esterase inhibitor that effectively increases concentration of acetylcholine at the sites of cholinergic transmission.[20] The role of acetylcholine, cholinergic agonists and cholinesterase inhibitors, collectively termed cholinomimetics, in modulation of pain and analgesia has been established.[21] Intraarticular injection of atropine-reversed thermal analgesia induced by intrathecal injection of neostigmine in the rat inflamed knee joint model.[6] In hot plate and tail immersion tests of nociception in rats, intrathecal administration of neostigmine, physostigmine and echothiophate produced antinociception.[22] Intrathecal injection of neostigmine reduced incision-induced mechanical hyperalgesia in rats.[23] These studies have focused on the role of cholinesterase inhibitors in modulating pain at local peripheral and spinal cord levels. Cholinomimetic agents also influence pain perception at the brain level. Intra-hippocampal microinjection of acetylcholine and pilocarpine decreased frequency of discharge of pain-exited neurons, and increased frequency of discharge of pain-inhibited neurons in the dorsal hippocampus in rats. Intra-hippocampal injection of atropine produced opposite effects.[24] Microinjection of cholinergic agonist, carbachol, into the intralaminar nucleus parafascicularis of thalamus produced antinociception in rats. Atropine reversed antinociception induced by carbachol[25] Cholinergic agonist carbachol and cholinesterase inhibitor physostigmine reduced the amplitude of evoked inhibitory and excitatory currents in the periaqueductal gray, and this effect was abolished by atropine.[26]

In the present study, physostigmine through muscarinic cholinergic receptors attenuates both phases of formalin-induced pain. The advantage of formalin test model is that it may provide a tool for studying the effects of pain and analgesia modulating agents for two types (phasic and tonic) of pain at a time.[27,28] Intrathecal injection of neostigmine reduced the time spent licking plus elevating the injected paw in the first phase (phasic pain) of formalin (5%, 50 μl)-induced pain in rats. The second phase (tonic pain) was slightly reduced only by higher dose of neostigmine.[12] Moreover, intrathecal injection of physostigmine, neostigmine and edrophonium produced a dose-dependent suppression of flinching in both phases of formalin-induced pain in rats. Intrathecal pretreatment with atropine prevented the suppressive effects of cholinesterase inhibitors on both phases of pain.[13–15] However, the absence of a descending cholinergic projection to the spinal cord in rats was reported.[29] Therefore, it seems that atropine-dependent antinociceptive effect of physostigmine observed in present study may be related to action of physostigmine on brain structures involved in pain perception.

In conclusion, the present data showed that central physostigmine suppressed both neurogenic (acute) and inflammatory (tonic) phases of formalin-induced pain. Atropine, a muscarinic receptor antagonist, prevented physostigmine-induced antinociception. Hence, brain cholinergic system through muscarinic receptors may be involved in modulation of pain.
